# The mediating role of psychological flexibility in the relationship between adverse childhood experiences and symptoms of depression, anxiety, and stress among Vietnamese college students

**DOI:** 10.1007/s44192-025-00293-4

**Published:** 2025-11-03

**Authors:** Truong Vuong Vu, Bao-Tran Nguyen-Duong, Ha Thi Thu Le, Tuan Van Nguyen, Vy Truc Le

**Affiliations:** 1 Ha Long University, Quang Ninh Province, Vietnam; 2 Ho Chi Minh City University of Education, Ho Chi Minh City, Vietnam; 3 Hong Duc University, Thanh Hoa Province, Vietnam; 4 Ha Noi Metropolitan University, Hanoi, Vietnam

**Keywords:** Adverse childhood experiences (ACEs), Psychological flexibility, Mental health, College students

## Abstract

Adverse childhood experiences (ACEs) are known as risk factors that contribute to mental health problems such as anxiety, depression, and stress in adulthood. Notably, the specific mechanisms underlying this association have been identified in only a limited number of studies. The present study aimed to explore the relationship between ACEs, psychological flexibility (PF), and mental health (anxiety, stress, and depression) in college students. A cross-sectional study was conducted among 302 college students in southern Vietnam. The data were analyzed using PLS-SEM to examine the relationship among the variables, and T-tests and One-way ANOVA in SPSS were employed to explore group differences. The results showed that PF functioned as a mediating factor in the pathway from ACEs to mental health. In addition, we found significant gender differences in PF, differences in mental health problems based on sources of emotional support, and differences in depression levels across religions. These findings provide valuable insights for both research and clinical practice in Vietnam, by highlighting PF as a crucial factor for future mental health interventions.

## Introduction

Adverse childhood experiences (ACEs) are traumatic events that occur before the age of 18 and are commonly categorized into three main groups: abuse, neglect, and family dysfunction [1]. These early traumas have long-term implications, manifesting in mental health disorders, addictions, and medical conditions throughout adulthood [2]. According to Tran, Dunne [3], ACEs are positively associated with adverse health outcomes and negatively associated with positive mental health. While there have been studies indicating the relationship between ACEs and mental health in Vietnam, it is worth noting that relatively few studies have investigated the specific mechanism underlying this association. Among current psychological theories, psychological flexibility (PF) within Acceptance and Commitment Therapy (ACT) offers a notable framework for explaining many mental health issues.

### Psychological flexibility in the context of acceptance and commitment therapy

Acceptance is the conscious adoption of an open, receptive, flexible, and nonjudgmental stance toward moment-to-moment experience [4]. According to Hayes, Luoma [5], acceptance involves actively and consciously embracing personal experiences without employing unnecessary defenses to alter their frequency or form. Acceptance does not require abandoning all activities or vividly recalling every memory that arises. Rather, it entails an active and flexible process of recognizing and observing psychological events as they occur, even when they intensify, thereby allowing these events to be engaged with more meaningfully [6]. Moreover, ACT shares similarities with other therapies that emphasize behavior modification in general, particularly in its focus on committed action toward behavioral change in accordance with chosen life values, which defines commitment [5]. This commitment involves taking proactive steps to live in alignment with values, even in the presence of discomfort or adversity [5].

Over the years, mental health issues have been examined from various theoretical perspectives within psychology. PF is a central concept in ACT, has been recognized as a transdiagnostic approach that explains many mental health problems and promotes human functioning [7–9]. According to ACT theory, the aim of this psychotherapy is to cultivate PF, which refers to enhancing individuals’ capacity to fully engage with the present moment and to either persist with or modify behaviors so that they are adaptive and aligned with personal values [5]. The PF model comprises six core therapeutic processes: acceptance, cognitive defusion, self as context, contact with the present moment, identifying with personal life values, and transforming values into committed action [5]. PF is demonstrated when individuals accept emotions, whereas its opposite is avoidance. Acceptance of thoughts and emotions becomes particularly difficult under stressful events, leading individuals to rely on avoidance strategies that heighten distress through rigid attempts to control experiences, ultimately resulting in maladaptive behaviors. Similarly, the antithesis of committed action is inaction, impulsivity, or avoidant persistence, which hinders individuals from engaging in adaptive behaviors and acting in accordance with their chosen values. Because PF is both a construct and a process, emphasizing acceptance and committed action does not mean that the remaining four components are unimportant; rather, these processes are interconnected and function together [4–6]. Previous studies have indicated that emotional and thought suppression, as well as experiential avoidance, are significant predictors of mental health outcomes and contribute to the development of psychological disorders [10, 11]. Reducing the counter-processes of the PF model may therefore lead to improved mental health. According to Hayes, Luoma [5], greater PF is associated with reduced symptoms of anxiety and depression.

In addition, we examined differences in PF across demographic factors. Rovner, Sunnerhagen [12] found that, when experiencing the same level of pain, women reported higher activity levels, greater pain acceptance, and stronger social support than men. Similarly, in the context of affective pain, acceptance-based coping strategies appear to be more suitable and beneficial for women [13].

### The role of psychological flexibility between adverse childhood experiences and mental health problems

Psychological inflexibility is a risk factor associated with various mental health issues [14–16]. The mediating role of PF in the relationship between ACEs and mental health has been widely discussed in the literature. For example, Taşören [17] reported that acceptance and action mediated the impact of childhood maltreatment on symptoms of stress, anxiety, and depression. Similarly, Baugh, Cox [18] indicated that PF mediated the relationship between childhood emotional maltreatment and beliefs in one’s romantic partner. In addition, mental health problems such as somatization [19], depression, and anxiety [8] have been shown to be influenced by ACEs through the mediating role of psychological inflexibility. Previous studies also showed that specific components of PF, such as mindfulness, which is considered a core approach of PF, mediated the relationship between sexual assault and depressive symptoms [20]. Furthermore, both experiential avoidance and behavioral passivity have been found to mediate the link between ACEs and severe mental health conditions, including depression [8, 21].

Additionally, we examine demographic factors such as religion and sources of support in relation to how individuals experience and cope with mental health challenges. Previous studies have found that some young adults avoid seeking professional psychological help [22], instead rely on non-professional sources such as family and friends, or on themselves [23–25]. Moreover, religious beliefs can serve as a source of psychological support during struggling times [26–30].

### Vietnam context

Vietnam is an Asian country with a diverse cultural heritage and historical influences from Confucianism, which have shaped aspects of its socioeconomic development. These values include traditional beliefs supporting patriarchal norms and accepting physical force within the home [31, 32]. Such cultural factors may contribute to an increased risk of specific ACEs. Previous study has found that the most commonly reported ACEs were emotional abuse, physical abuse, and witnessing household violence [3]. Studies have also indicated that youth in low- and middle-income countries (LMICs) are more likely to experience ACEs than their peers in high-income countries (HICs) due to complex living conditions [33, 34]. Among Vietnamese young people, ACEs have been associated with lower educational attainment, methamphetamine use, buying sex, depression, psychotic symptoms, and a reported need for mental health support [35]. Therefore, exploring ACEs and their relationship with mental health in Vietnam is essential to contributing to the existing body of data on ACEs in LMICs.

On the other hand, Vietnam’s national circumstances and social context also pose significant challenges for clinical practitioners. According to Giebel, Gabbay [36], stigma, limited mental health literacy, the burden of illness in low- and middle-income countries, cultural barriers to care and practical difficulties in delivering therapies all intensify these challenges [36]. We chose the ACT concept of PF as the foundation for examining research dimensions and therapeutic practices in Vietnam. ACT, a third-wave intervention that evolved from cognitive behavior therapy (CBT), integrates acceptance, mindfulness, and behavioral activation strategies to enhance PF and support individuals in engaging in value-consistent behavior [37]. The elements of acceptance and mindfulness are closely related to Asian philosophies such as Hinduism and Buddhism, where acceptance is oriented toward enlightenment and detachment from the world [38]. In therapeutic contexts, however, acceptance is directed toward alleviating negative symptoms [38]. Therefore, the purpose of this study is to examine the role of PF as a mediating mechanism in the relationship between ACEs and common mental health symptoms, including stress, anxiety, and depression. In addition, we explore the contribution of other factors, such as gender, religion, and sources of emotional support in shaping the impact of ACEs on mental health.

Derived from these discussions, Hypotheses 1, 2, and 3 are proposed for this study.

#### Hypothesis 1

*Acceptance and action (AAQ) would mediate the relationship between Adverse childhood experiences (ACEs) and symptoms of anxiety*.

#### Hypothesis 2

*Acceptance and action (AAQ) would mediate the relationship between Adverse childhood experiences (ACEs) and symptoms of depression*.

#### Hypothesis 3

*Acceptance and action (AAQ) would mediate the relationship between Adverse childhood experiences (ACEs) and symptoms of stress*.

## Materials and methods

### Participants

This study included a sample of 302 participants and aimed to examine the relationship between demographic variables and psychological outcomes. The demographic characteristics assessed were gender, residential area, religion, and sources of emotional support. Descriptive statistics for the main variables are presented in Table 1**.**

**Table 1:** Demographic characteristics table

	Total (n = 302)	AAQ-II		Depresion		Anxiety		Stress	
	Frequency (%)	M ± SD	*p*	M ± SD	*p*	M ± SD	*p*	M ± SD	*p*
Gender^a^			< 0.05		> 0.05		> 0.05		> 0.05
Male	127 (42.1)	2.24 ± 1.41		0.84 ± 0.69		0.88 ± 0.64		1.06 ± 0.68	
Female	175 (57.9)	2.68 ± 1.44		0.92 ± 0.70		0.92 ± 0.67		1.14 ± 0.67	
Residence^a^			< 0.05		> 0.05		> 0.05		< 0.05
City	156 (51.7)	2.71 ± 1.39		0.88 ± 0.65		0.93 ± 0.67		1.19 ± 0.68	
Countryside	146 (48.3)	2.26 ± 1.46		0.89 ± 0.74		0.88 ± 0.64		1.02 ± 0.65	
Religion^b^			> 0.05		< 0.001		< 0.05		< 0.05
Catholicism	34 (11.3)	2.78 ± 1.51		1.22 ± 0.77		1.18 ± 0.81		1.29 ± 0.78	
Buddhism	64 (21.2)	2.60 ± 1.47		0.94 ± 0.77		0.84 ± 0.64		1.09 ± 0.73	
Other Religions	3 (1.0)	3.48 ± 1.41		2.00 ± 1.00		1.81 ± 0.73		1.90 ± 0.86	
No Religion	201 (66.6)	2.40 ± 1.41		0.79 ± 0.62		0.87 ± 0.62		1.07 ± 0.64	
Source of emotional support options while encountering struggles^b^			< 0.05		< 0.05		> 0.05		< 0.05
Family	105 (34.8)	2.08 ± 1.39		0.78 ± 0.69		0.86 ± 0.71		0.97 ± 0.65	
Friend	43 (14.2)	2.50 ± 1.29		0.77 ± 0.63		0.85 ± 0.59		1.15 ± 0.67	
Formal help (Psychologist, Psychiatrist,…)	0 (0)	–		–		–		–	
Overcome by yourself	154 (51.0)	2.78 ± 1.46		0.99 ± 0.70		0.95 ± 0.64		1.19 ± 0.67	

^a^Independent Sample t-test; ^b^One-way Anova

Acceptance and Action (AAQ-II)

### Procedure

This cross-sectional study focused on young adults who were undergraduate students in southern Vietnam. The sample size was determined based on recommendations suggesting that a minimum of 100 to 200 participants is appropriate for studies involving path estimation, particularly when using structural equation modeling. Data were collected from August to October 2023 through an online Google Form survey, employing a random sampling method. An attention-check question was included as a screening criterion, and responses failing to meet this requirement were excluded from the final dataset to ensure data reliability. Informed consent was obtained from all participants, with anonymity and confidentiality assured. Participants were informed of their obligations and their right to withdraw from the study prior to participation. They were asked to provide socio-demographic information, including gender, residence, religion, and sources of emotional support during struggling times. The questionnaire consisted of three measures: (1) The Adverse Childhood Experience (ACE-Q) 10-item version; (2) Acceptance and Action Questionnaire-II-7 (AAQ); and (3) Depression Anxiety Stress Scales (DASS-21).

### Measures

Three instruments were chosen: (1) The Adverse Childhood Experiences (10-item version); (2) Acceptance and Action Questionnaire-II-7; (3) Depression Anxiety Stress Scales (DASS-21). The scales were translated into Vietnamese for use in this study. The forward translation (from English to Vietnamese) was conducted by a native Vietnamese speaker fluent in English, while the backward translation (from Vietnamese back to English) was carried out by a native English speaker fluent in Vietnamese. The research team then carefully compared the original English version, the translated Vietnamese version, and the back-translated English version to identify and resolve any discrepancies, ensuring both linguistic accuracy and conceptual equivalence.

- *The Adverse Childhood Experiences Questionnaire (ACE-Q 10-item)*: Studies on Adverse Childhood Experiences (ACEs) have been conducted over an extended period, yielding substantial contributions to the field. Notably, the Adverse Childhood Experiences Questionnaire (ACE-Q) has served as a foundational instrument underpinning a wide range of subsequent research [39]. The ACE-Q is a 10-item self-reported questionnaire developed by Finkelhor, Shattuck [40]. It encompasses areas such as abuse (e.g., “Did a parent or other adult in the household often… swear at you, insult you, or put you down?”), neglect (e.g., “Did you often feel that no one in your family loved you or thought you were important or special?”), and household dysfunction (e.g., “Was a household member depressed or mentally ill, or did a household member attempt suicide?”). Respondents reply with “Yes” or “No” to each inquiry. The original research indicates that the ACE-Q possesses exceptional psychometric qualities, featuring a strong one-factor structure and good internal consistency. Previous studies conducted in Vietnam have utilized this questionnaire among young adult samples to investigate its association with mental health [35].
- *The Acceptance and Action Questionnaire-II-7 (AAQ-II-7)*: The AAQ-II-7 is a seven-item scale developed by Bond, Hayes [41] to assess PF, namely the capacity to accept adverse events and pursue values-driven actions. Items encompass declarations like “I’m afraid of my feelings” and “I worry about not being able to control my thoughts and feelings”. Responses are evaluated using a 7-point Likert scale, with “1 = never true” and “7 = always true”. Elevated scores signify diminished PF. The reliability of the AAQ-II-7 scale has been reported as 0.87 in original studies and demonstrated strong internal consistency and dependability, with Cronbach’s α values ranging from 0.90 to 0.92. The AAQ-II-7 scale showed a reliability coefficient of 0.92 in this study.
- *The Depression Anxiety and Stress Scales (DASS-21)*: The DASS-21 is a 21-item instrument intended to assess the conditions of depression, anxiety, and stress [42]. Items include statements such as “I found it difficult to relax” (stress), “I felt that I had nothing to look forward to” (depression), and “I experienced trembling” (anxiety). Each subscale consists of seven items. Responses are evaluated using a 4-point Likert scale, with ratings from “0 = did not apply to me at all” to “3 = applied to me very much, or most of the time.” The aggregate score for each subscale is doubled to correspond with the complete 42-item version. Original investigations have indicated a reliability range of 0.81 to 0.91 for the subscales. In our study, Cronbach’s alpha varied from 0.84 to 0.89 for the subscales.

### Analytic approaches

The data purification and coding processes were conducted in Excel. A descriptive analysis of univariate and bivariate data was performed to characterize individuals and examine the distribution of variables across groups. In addition, independent sample t-tests and one-way ANOVA were used to examine group differences in AAQ, stress, anxiety, and depression. The Statistical Package for the Social Sciences (SPSS) version 26.0 was utilized for this phase.

We executed a mediated path model with ACE as the input variable and stress, anxiety, and depression as output variables, while AAQ serving as the mediating variable. To begin with, the necessary criteria for reflective latent constructs included indicator reliability (outer loading), construct reliability (Cronbach’s alpha—CA, composite reliability—CR), convergent validity (average variance extracted—AVE), and discriminant validity (HTMT criterion). The structural equation model was assessed using multicollinearity (variance inflation factor—VIF), coefficient of determination (R^2^), predictive relevance (Q^2^), effect sizes (f^2^), and the significance and relevance of path coefficients. A comprehensive PLS-SEM analysis based on 5000 bootstrap samples was employed to calculate path coefficients along with P values and specific indirect, specific direct, and total effects. Subsequently, the data in this study were analyzed using Smart Partial Least Squares Structural Equation Modeling (Smart PLS-SEM), a variance-based approach, in the latest version of SmartPLS 3 (3.2.6). Due to reflective measurement models, mediating hypotheses, and normally distributed data, PLS-SEM is chosen for data analysis.

### Ethical aspects

This study was conducted as part of an undergraduate thesis project. According to our institutional regulations, such projects are exempt from formal IRB approval, provided that they strictly comply with institutional and international IRB guidelines. The study complies with the ethical principles and standards for research in the social sciences and humanities, as promulgated in Decision No. 4111/QĐ-ĐHSP dated December 27, 2022, by the Rector of Ho Chi Minh City University of Education. Additionally, the study was conducted under the Declaration of Helsinki and adhered to the principles outlined by the American Psychological Association.

## Results

### Descriptive analysis

Table 2 indicates the proportion of college students who reported at least one ACE-related incident. Among the ten categories of ACEs, the most commonly reported were parental separation (12.3%), emotional neglect (11.9%), and emotional abuse (9.6%). The least common ACEs reported were incarcerated household members (1.7%), physical neglect (4.0%), and household physical violence (4.3%). Among these categories of adverse experiences described, 21.2% of participants had only one type of ACE, 10.3% had two types of ACE, 3.6% had three types of ACE, and 3.6% had four ACEs. Meanwhile, 61.3% of subjects reported no ACEs. The results are shown in Table 3**.**

**Table 2:** Descriptive statistics of types of adverse childhood experiences

Category of childhood adversity	Frequency (%)
Emotional abuse	29/302 (9.6)
Physical abuse	20/302 (6.6)
Sexual abuse	15/302 (5.0)
Emotional neglect	36/302 (11.9)
Physical neglect	12/302 (4.0)
Parental separation	37/302 (12.3)
Household physical violence	13/302 (4.3)
Household substance abuse	19/302 (6.3)
Household mental illness	25/302 (8.3)
Incarcerated household member	5/302 (1.7)

**Table 3:** Adverse childhood experiences total score

0 ACE	185 (61.3)
1 ACE	64 (21.2)
2 ACEs	31 (10.3)
3 ACEs	11 (3.6)
from 4 to 10 ACEs	11 (3.6)
Total	302 (100)

### Normality and comparing tests

According to Mishra, Pandey [43], a sample size greater than 300 demonstrates adequate normalcy when absolute skewness and kurtosis values of ≤ 2 and ≤ 4, respectively. The present study included 302 individuals, and the skewness and kurtosis values met these thresholds. These assessments confirmed the normal distribution of the dataset, thereby enhancing the reliability and validity of subsequent statistical analyses. Consequently, parametric tests were applied to the AAQ scale as well as the three subscales of DASS-21: Anxiety, Depression, and Stress.

With T-test analysis, there was a significant difference in AAQ between males (M = 2.24; SD = 1.41) and females (M = 2.68; SD = 1.44); (t(275.515) = − 2.622, *p* < 0.05). In this study, we found a significant difference in the level of depression when considering sources of emotional support options (F(2,299) = [3.549], *p* < 0.05). Tukey’s HSD Test for multiple comparisons found that college students who opt for family as a source of emotional support (M = 0.78; SD = 0.69) have lower levels of depression than college students overcome by themselves (M = 0.99; SD = 0.70) (*p* < 0.05, 95% CI = [0.003, 0.41]). We continued to consider levels of stress among college students with sources of emotional support options, and the results showed a statistically significant difference (F(2,299) = [3.565], *p* < 0.05). Specifically, there was a significant difference between individuals who chose family (M = 0.97; SD = 0.65) with lower levels of stress than individuals who chose to overcome by themselves (M = 1.19; SD = 0.67) (*p* < 0.05, 95% CI [0.02, 0.42]).

We continued to examine the difference in religious variables and found a statistically significant difference in levels of depression (F(3,298) = [6.830], *p* < 0.001). Tukey’s HSD Test for multiple comparisons revealed that there was a significant difference in levels of depression between college students who were involved in Catholicism (M = 1.22; SD = 0.77) and those who had no religion (M = 0.79; SD = 0.62) (*p* < 0.05, 95% CI = [0.10, 0.74]). In addition, the levels of depression have a significant difference between college students who were involved in Buddhism (M = 0.94; SD = 0.77) and those who were in other religions (M = 2.00; SD = 1.00), (*p* < 0.05, 95% CI = [− 2.08, − 0.03]). We also found that people with other religions (no Catholicism and Buddhism) (M = 2.00; SD = 1.00) have greater levels of depression than those who had no religion (M = 0.79; SD = 0.62) (*p* < 0.05, 95% CI = [0.19, 2.21]).

### Model specification

**Fig. 1:**
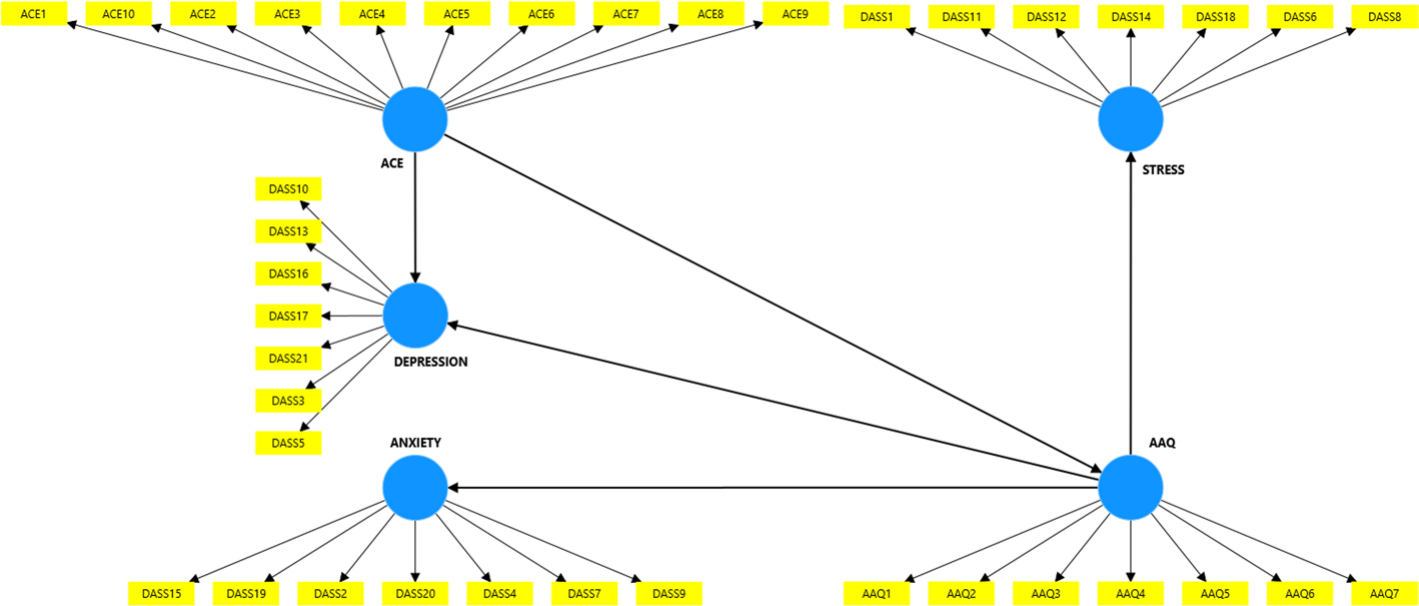
Partial least squares structural equation modeling (PLS-SEM). *Note*: ACE (Adverse Childhood Experience), AAQ (Acceptance and Action), Stress, Anxiety, and Depression are subscales of DASS-21

The final PLS model is illustrated in Fig. 1. The research model proposed for this study comprises three distinct latent vectors: AAQ (which includes items from the Acceptance and Action Questionnaire), ACE (which contains items from the Adverse Childhood Experiences questionnaire), and ANXIETY, DEPRESSION, and STRESS (which comprise items from the DASS-21 scale).

### Measurement model

The outer loadings varied from 0.639 to 0.871 and were all statistically significant (*p* < 0.001). The components with loading below 0.70 were retained due to the appropriate reliability of these structures [44]. Table 4 presents the reliability and validity of the endogenous latent variables. The constructs’ average variance explained (AVE) ranges from 0.514 to 0.677. Cronbach’s alpha coefficients vary from 0.842 to 0.920. The CR coefficients ranged from 0.879 to 0.936, beyond the threshold of 0.7 [45], indicating that the variance shared among the individual indicators is substantial. The HTMT criterion is a sophisticated strategy recently introduced by Dijkstra and Henseler [46] and is extensively utilized. It is proposed that if the HTMT value for each pairwise construct does not exceed the threshold of 0.9, the discriminability of the reflective model is confirmed. The HTMT values are presented in Table 5.

**Table 4:** Reliability and validity statistics

Variables	Cronbach’s alpha (CA)	Composite reliability (CR)	Average variance extracted (AVE)
AAQ	0.920	0.936	0.677
Anxiety	0.842	0.879	0.514
Stress	0.867	0.898	0.559
Depression	0.893	0.916	0.612

**Table 5:** Discriminant validity assessment using the HTMT criterion

AAQ			
Anxiety	0.650 [0.561, 0.727]		
Stress	0.725 [0.643, 0.796]	0.991 [0.957, 1.024]	
Depression	0.690 [0.600, 0.764]	0.888 [0.824, 0.945]	0.902 [0.856, 0.942]

### Structural model

Before evaluating the structural model, it is essential to resolve the collinearity issue to reduce bias in the regression results. The inner values of all predictor variables varied from 1.000 to 1.181, consistently remaining below the threshold of 5.0 [47]. Consequently, we can ascertain that collinearity is not a concern in the present model (Table 6).

**Table 6:** Collinearity statistics

VIF	
AAQ → Anxiety	1.000
AAQ → Depression	1.181
AAQ → Stress	1.000
ACE → AAQ	1.000
ACE → Depression	1.181

The study employed the bootstrapping technique with 5,000 subsamples to examine the path coefficient. Table 7 demonstrated a significant positive influence of acceptance and action (β = 0.578, *p* < 0.001, 95% CI [0.478, 0.659]), as well as adverse childhood experiences (β = 0.476, *p* < 0.001, 95% CI [0.080, 0.923]) on depression. Furthermore, Table 7 demonstrated that acceptance and action had a significant positive influence on anxiety (β = 0.613, *p* < 0.001, 95% CI [0.545, 0.683]) and stress (β = 0.661, *p* < 0.001, 95% CI [0.592, 0.730]). The data indicated that adverse childhood experiences positively influenced acceptance and action (β = 1.416, *p* < 0.001, 95% CI [1.129, 1.790]).

**Table 7:** Evaluation of structural model and hypothesis testing

Path	β	t	*p*	95% CI Lower	95% CI Upper
*Direct effects*					
AAQ → Anxiety	0.613	17.233	< 0.001	0.545	0.683
AAQ → Depression	0.578	12.564	< 0.001	0.478	0.659
AAQ → Stress	0.661	18.838	< 0.001	0.592	0.730
ACE → AAQ	1.416	8.308	< 0.001	1.129	1.790
ACE → Depression	0.476	2.240	< 0.05	0.080	0.923
*Indirect effects*					
ACE → AAQ → Anxiety	0.868	7.070	< 0.001	0.666	1.144
ACE → AAQ → Depression	0.819	6.972	< 0.001	0.611	1.077
ACE → AAQ → Stress	0.936	7.217	< 0.001	0.722	1.227

The findings indicated the presentation of indirect effects from adverse childhood experiences (β = 0.868, *p* < 0.001, 95% CI = [0.666; 1.144]) on anxiety through acceptance and action. Thus, hypothesis 1 was accepted. Furthermore, a significant effect of adverse childhood experience on depression (β = 0.819, *p* < 0.001, 95% CI = [0.611; 1.077]) was observed, validating the supplementary partial mediation role of acceptance and action; hence, hypothesis 2 was affirmed. Additionally, hypothesis 3 was confirmed, indicating that adverse childhood experiences exerted a significant indirect positive effect on stress through acceptance and action (β = 0.936, *p* < 0.001, 95% CI = [0.722; 1.227]) [48]. All effects were statistically significant at the 2.5% level, and the value of 0 was excluded from the 95% confidence intervals.

The effect size was highest for the pathway from acceptance and action to stress (f^2^ = 0.776, *p* < 0.001), signifying a substantial effect. The effect size for the relationship between adverse childhood experiences and acceptance and action was minimal (f^2^ = 0.181, *p* = 0.001), signifying a medium-level influence [49, 50].

The coefficient, adverse childhood experiences, and acceptance and action accounted for 41.1% of the variance in depression. Adverse childhood experiences accounted for 15.3% of the difference in acceptance and action. Additionally, the variables of acceptance and action accounted for 37.6% of the variance in anxiety and 43.7% of the variance in stress. The adjusted R^2^ ranged from 15.0 to 43.5%, indicating a weak to moderate level of predictive accuracy [51, 52].

To evaluate the predictive relevance of the model, we employed the blindfolding process with a predetermined distance of 7. The Q^2^ outcomes for all endogenous constructs exceeded 0, ranging from 0.019 to 0.086, for adverse childhood experiences, acceptance and action, and all three types of mental health. Therefore, in the present model, predictive significance was firmly proven. All theories were substantiated by empirical evidence (Table 8).

**Table 8:** Structural model estimates

Construct	R^2^	Q^2^ Predict
AAQ	0.153	0.086
Anxiety	0.376	0.019
Stress	0.437	0.042
Depression	0.411	0.066

## Discussion

The primary objective of the study was to examine whether PF mediates the relationship between ACEs and symptoms of stress, anxiety, and depression. The results showed that PF, particularly its acceptance and action components, played a mediating role, highlighting its importance as a primary focus for intervention. Additionally, the study found that females reported higher AAQ-II scores than males, indicating lower levels of PF demonstrated higher PF than males. Participants with familial support reported lower levels of stress and depression, whereas those relying on self-coping showed higher levels of both. Differences in depression levels were also observed across religious groups. These findings not only validate the significance of the ACT-based approach but also emphasize the necessity of culturally adapted mental health therapies in Vietnam.

### Gender differences in psychological flexibility

According to our findings, females demonstrated greater AAQ-II scores compared to males, indicating lower levels of PF. This does not fully aligns with a prior study indicating that, when experiencing the same pain, women show a higher level of activity and acceptance than men [12]. Nevertheless, ACT interventions targeting depressed women with breast cancer have been shown to enhance both PF and acceptance of pain [53]. Therefore, such interventions may still be effective for women, helping to enhance PF in those with lower levels. Similarly, Stinson, Lasker [54] reported that following pregnancy loss, women tend to experience more sadness, whereas men are more likely to suppress their emotions due to cultural norms. Such denial or avoidance reflects a lower degree of PF, which often poses challenges in psychotherapy with male clients. This issue is particularly evident in many Asian societies, where masculinity and strength are strongly emphasized. Our results indicate that males exhibited higher levels of psychological flexibility (PF), and also suggest that promising ACT-based interventions may be effective even for men.

### Differences among sources of support in depression and stress

Our findings indicated that college students who primarily seek support from families when facing emotional difficulties report lower levels of depression and stress compared to those who attempt to cope alone. This aligns with a previous study showing that family remains the main source of support for mental health concerns [23]. Manczak, Skerrett [55] similarly found that family support protects young adults from the negative effects of life stress. Moreover, family support has been shown to have a stronger impact on reducing depression, particularly among older adults and within Asian communities [56]. These results are noteworthy in the Asian context, where family is regarded as the primary source of care during illness [23]. This cultural orientation may also explain why none of the participants in this study sought professional help. Chen, Xu [23], reported that healthier family functioning is associated with a lower likelihood of seeking professional services. Conversely, young adults who rely solely on themselves to manage emotional distress may experience greater depression and stress, possibly due to barriers such as stigma and negative attitudes toward professional help-seeking [57]. In Vietnam, this problem is compounded by relatively low levels of mental health literacy compared to other countries [58]. Therefore, enhancing public awareness and integrating mental health screening into primary care services is essential.

### Differences in depression by religious aspect

Our findings indicated significant differences in depression levels across religious groups, including Catholicism, Buddhism, other religions, and those without a religion. While prior studies consistently report lower rates of depression among individuals who regularly attend church, particularly Roman Catholics [59], our results showed the opposite pattern: Catholics in our sample reported higher levels of depression compared to those with no religious affiliation. According to Exline, Yali [60], despite the comfort religion can provide, religious strain may also arise, which is associated with increased depression and suicidality. Similarly, previous studies have shown that unmet religious expectations may contribute to guilt and despondency [61]. In contrast, we found that Buddhist participants reported lower depression levels than individuals affiliated with other non-Catholic religions. Wilken and Miyamoto [62] reached a similar conclusion, showing that Buddhist teachings provide practitioners with emotion regulation strategies that predict lower depressive symptoms compared to Protestants. Such strategies, which emphasize awareness and acceptance of emotions, align closely with the PF model underlying ACT. These findings suggest that ACT-based interventions may be particularly suitable for the Buddhist populations in Vietnam.

### The relationship between adverse childhood experiences, psychological flexibility, and symptoms of anxiety, depression, and stress

The present study confirmed the mediating role of PF in the relationship between ACEs and mental health problems. Specifically, the association between ACEs and symptoms of anxiety, depression, and stress was mediated by PF, as measured by the AAQ. These findings are consistent with ACT theory and previous studies. For example, Taşören [17] reported that psychological inflexibility, assessed with AAQ-II, mediated the relationship between childhood maltreatment and symptoms of depression, anxiety, and stress. Similarly, Makriyianis, Adams [8] discovered that ACEs were associated with anxiety and depression, with psychological inflexibility serving as the mediator. Evidence from more specific contexts also aligns with our results. Rosenthal, Hall [21] found that women with childhood sexual abuse histories tend to avoid unpleasant internal experiences, which contributes to later psychological distress. Likewise, another study on bereaved individuals demonstrated that avoidance mediated the relationship between rumination and depression [63]. Among college students, inaction has also been identified as a mediating factor between ACEs and depression [8]. Taken together, these findings highlight avoidance and inaction as key components of psychological inflexibility that mediate the impact of ACEs on mental health problems.

### Limitations and future directions

On one hand, this study contributes novel insights with implications for both research and clinical practice; on the other hand, it also presents certain limitations. First, the data were collected through self-report questionnaires, which are subject to potential bias and may compromise reliability. Future research studies could employ a longitudinal design or in-depth qualitative methods to address this issue. Second, as the study adopted a cross-sectional design, causal relations between variables cannot be established. Third, although random sampling was used, the participants were drawn solely from universities in southern Vietnam, which restricts the representativeness of the sample. Finally, while the sample of 302 observations was adequate for statistical analysis, it remains relatively modest, thereby limiting statistical power and the generalizability of the findings. Future studies should therefore employ larger and more diverse samples, together with rigorous methodologies, to strengthen the robustness and applicability of the results.

This study examined the relationship between ACEs, PF, and mental health outcomes. However, because ACEs were explored as a general construct, the findings do not provide detailed insights into specific categories such as abuse, neglect, and family dysfunction. Future studies should therefore investigate the distinct pathways linking each ACE category with PF and mental health in order to maximize the value of the ACE questionnaire. Moreover, this study confirmed the mediating role of PF, suggesting that enhancing flexibility may reduce the adverse impact of ACEs on anxiety, depression, and stress. Future studies should examine the feasibility and clinical effectiveness of ACT as an intervention to strengthen PF.

### Implications

To our knowledge, this is the first study examining the relationship between ACEs, PF, and mental health (anxiety, stress, and depression) in Vietnam. The findings provide valuable implications for both research and clinical practice, underscoring PF as a crucial factor in mitigating the adverse effects of ACEs. These results also suggest that clinicians and counselors may consider ACT, which has been described as a promising trauma event intervention offering a non-pathologizing approach that supports clients in building fulfilling lives despite persisting distress [64]. Furthermore, exploring PF in the Vietnamese context contributes to a broader understanding of Asian populations, where cultural similarities may shape coping mechanisms. For example, our findings confirmed that females reported greater AAQ-II scores than males and that families serve as a source of support during adversity. These results may reflect cultural values that emphasize family involvement in illness and reinforce masculine norms discouraging emotional expression; however, ACT-based interventions may hold promise for supporting male participants. Finally, the observed differences by religion provide additional perspectives on how spiritual orientation may shape mental health outcomes.

## Conclusion

The primary finding of this study was the vital role of PF as a mediator between adverse childhood experiences (ACEs) and mental health outcomes, such as anxiety, depression, and stress, in Vietnamese college students. The findings also highlight the influence of gender, familial support, and religious affiliation on mental health, emphasizing the need for culturally sensitive approaches to mental health care. By validating the applicability of Acceptance and Commitment Therapy (ACT) within the Vietnamese context, this research advocates for integrating PF-focused interventions into clinical and community settings. Despite limitations such as reliance on self-reported data and a cross-sectional design, the study provides foundational insights for advancing mental health research and practice, offering pathways to improve support systems and therapeutic outcomes in Vietnam and similar cultural contexts.

## Data Availability

The raw data collected and analysed in this study are not publicly available due to ethical and confidentiality considerations, in accordance with the regulations of the approving institution. Researchers who wish to access the data for reference may contact the corresponding author or one of the co-authors. Any potential data sharing will be subject to discussion with the institution and considered on a case-by-case basis. While we support the principles of open access and data transparency, participant privacy and ethical obligations prevent us from depositing the raw data in a public repository.

## Appendix

**Table:**

Latent variable correlation matrix					
	AAQ	ACE	Anxiety	Depression	Stress
AAQ	1.000	0.391	0.613	0.630	0.661
ACE	0.391	1.000	0.220	0.358	0.274
Anxiety	0.613	0.220	1.000	0.792	0.874
Depression	0.630	0.358	0.792	1.000	0.800
Stress	0.661	0.274	0.874	0.800	1.000

All correlations are significant at *p* < 0.001
